# Direct-Acting Antivirals in the Treatment of Hepatitis C Virus (HCV)/Human Immunodeficiency Virus (HIV) Co-infected Patients: Real-Life Experience From Nepal

**DOI:** 10.7759/cureus.15932

**Published:** 2021-06-26

**Authors:** Sudhamshu KC, Niyanta Karki, Dilip Sharma, Sandip Khadka, Pratap S Tiwari

**Affiliations:** 1 Hepatology, National Academy of Medical Sciences, Kathmandu, NPL

**Keywords:** direct acting antivirals, chronic hepatitis c, hiv-hcv co-infection, real life experience, nepal

## Abstract

Background

Direct-acting antivirals (DAA) have revolutionized the treatment of chronic hepatitis C patients. However, the real-life data regarding its use in a human immunodeficiency virus (HIV) co-infection from a developing country is lacking. We aimed to see the efficacy of DAA in hepatitis C virus (HCV)/HIV co-infected populations.

Methods

In this prospective, observational, intention-to-treat study from Nepal, treatment-naïve patients undergoing treatment for chronic HCV in HIV co-infected individuals with DAA were studied. Patients on nevirapine were switched to efavirenz or atazanavir. Patients received sofosbuvir/ledipasvir or sofosbuvir/daclatasvir with or without ribavirine. Sustained virological response (SVR) at week 12, adverse events, and treatment compliance were evaluated. Treatment efficacy was compared between cirrhotic and non-cirrhotic patients.

Results

Of 218 patients presenting with an anti-HCV report, 181 (83%) had detectable HCV RNA. Eighty-five (85; 47%) patients were having ART at presentation. Three patients could not complete treatment due to gall stone pancreatitis and 82 completed treatment. Twenty-nine (29; 35%) were cirrhotic at presentation. Fifty-one (51; 62%) patients were genotype 3, 27 (33%) were genotype 1, three (4%) were mixed 1a/3, and one (1%) was 6. Seventy-four (74; 90%) had SVR12. Non-cirrhotics had 96% SVR compared to 79% in cirrhotics. SVR in genotype 3 was 88% while it was 93% in genotype 1.

Conclusions

Real-life experience showed that the DAAs are equally effective in HCV HIV co-infected patients. In non-cirrhotic patients, the result is comparable to mono-infected patients. Genotype 3 co-infected are also difficult-to-treat patients. DAA treatment is well-tolerated in HCV/HIV co-infected patients, and there was no dropout during treatment.

## Introduction

It is estimated by World Health Organization that there are 35 million people living with human immunodeficiency virus (HIV) globally [1]. The same report stated that approximately 25% or 8.75 million are coinfected with hepatitis C virus (HCV). On the other hand, the 8.75 million people with HIV/HCV coinfection constitute only 6%-11% of the 80 to 135 million people estimated to be HCV-infected globally [2-3]. HIV-HCV coinfected patients experience early mortality due to liver disease, and this represents the main cause of mortality in this subgroup of patients [4-7].

The potent and efficacious direct antiviral agent (DAA), which is well-tolerated, has drastically changed the prognosis of chronic hepatitis C (CHC) treatment. It is estimated that compared to HIV-negative populations, the risk of HCV infection is six times higher for HIV-positive individuals [1]. The prevalence of HIV/HCV coinfection varies widely by geography and demography. However, it is consistently high among people who inject drugs (PWID) [8-9].

Nepal is among the low prevalence country of HCV-infected individuals. The prevalence is less than 1% in different studies [10-11]. It has been estimated that 50,000 individuals (approximately 0.3% of the total adult population) inject drugs in Nepal with a significant increase in this figure occurring in recent years [12]. The prevalence of HCV infection in the drug-users community is 80%-85% [13-14]. We aimed to see the results of DAA treatment in HCV-HIV co-infected patients, which has not been published in our country so far.

## Materials and methods

This is a prospective, observational study between April 2015 and July 2017 from Nepal. A public call was made for enrollment for the treatment and a discount on various tests and medicine was announced. Treatment-naive patients with a history of intravenous (IV) drug abuse, presenting with a positive anti-HCV blood test report, who were co-infected with HIV were included in the study. An HCV RNA quantitative test was done by real-time PCR in all individuals. Those with detectable RNA were subjected to a regular blood test, genotyping of HCV RNA, ultrasound examination, and fibroscan (Echosense, France) after overnight fasting. Cirrhotic patients on ultrasound or patients with a fibroscan value of more than 12.5 kPa underwent upper gastrointestinal (GI) endoscopy examination. Those with ascites in ultrasound underwent ascitic fluid analysis, including adenosine deaminase (ADA), to rule out spontaneous bacterial peritonitis and tubercular peritonitis. If there was any suspicion of additional etiology of cirrhosis (other than viral and alcohol), other investigations (for example, antinuclear antibodies (ANA), serum immunoglobulin G (S IgG), iron profile, copper profile, etc.) were done.

For the treatment of HCV chronic hepatitis, two generic DAAs with or without ribavirine were used. For genotype (GT) 1, a fixed-dose combination of sofosbuvir 400 mg plus ledipasvir 90 mg was given for 12 weeks without ribavirine for chronic hepatitis and compensated LC patients. For decompensated liver cirrhosis (LC) weight-based ribavirine, 1000 mg for < 75 kgs and 1200 mg for > 75 kgs were given for the same period. For GT 3 patients and other GTs, a pan-genotypic combination of sofosbuvir 400 mg and daclatsvir 30 mg or 60 mg (depending upon the types of DAA used) was used for 12 weeks in non-cirrhotic patients and 24 weeks in cirrhotic patients. For cirrhotic patients, weight-based ribavirine, 1000 mg for < 75 kgs and 1200 mg for > 75 kgs was given for 24 weeks duration. The dose of ribavirine was adjusted as per the hemoglobin report. For more than 10 gm/dL, a full dose was given. Two hundred mg/day was reduced if it was between 8.5-10 mg/dL.

Patients on nevirapine were switched to efavirenz or atazanavir before the initiation of treatment to avoid drug-drug interaction. Priority was given to change to atazanavir, as the dose of daclatasvir required was only 30 mg OD. The dose of daclatasvir was adjusted accordingly. Sustained virological response at week 12 (SVR12, which is defined as no viral activity even after 12 weeks of treatment), adverse events, and treatment compliance were evaluated. Treatment efficacy was compared between cirrhotic and non-cirrhotic patients.

The schedule of visits after the initiation of treatment was days 14, 42, and 84. At every visit, the occurrence of adverse events related to the complications of the DAA treatment, antiretroviral (ARV) treatment, and treatment related to liver disease, if any, was collected and reported. Treatment changes were made accordingly as per standard protocol.

Data were put in Microsoft Excel (Microsoft Corporation, Redmond, WA) format, and statistical analysis was done using SPSS ver 23 software (IBM Corp., Armonk, NY). Written informed consent was taken from each patient before starting treatment. Institutional review board (IRB) approval was given by the IRB of Norvic International Hospital.

## Results

Two hundred eighteen presented with a positive anti-HCV positive report during the study period. One-hundred eighty-one (181; 83%) had detectable HCV RNA by polymerase chain reaction (PCR). Spontaneous HCV clearance was evident in 17% of subjects; those who tested positive for anti-HCV antibodies. Eighty-five (85) patients were taking ART at the time of presentation and were included in the study. A total of 21 patients had to change the ART regimen. The ART regimen before starting DAAs is shown in Table 1.

**Table 1:** ARV regimens at the time of presentation ZDV: zidovudine, 3TC: lamivudine, EFV: efavirenz, LPV: lopinavir, r: ritonavir, ATV: atazanavir, TDF: tenofovir, ARV: antiretroviral

ARV	(N=82)	%
TDF, 3TC, ATV/r	35	43%
ZDV, 3TC, EFV	18	22%
TDF, 3TC, EFV	17	21%
TDF/3TC/EFV	7	9%
TDF, 3TC, EFV	3	4%
TDF, 3TC, LPV/r	1	1%
ZDV, 3TC, LPV/r	1	1%
Total	82	100%

Three patients could not complete treatment due to gall stone pancreatitis and 82 completed treatment and were available for the final analysis.

Out of 82 patients treated, 78 patients were male and four patients were female showing male predominance. The majority of the patients were from 20-40 age groups, showing this disease as a disease of younger age group in our country. All but one patient had contracted the disease by sharing needles. One female had contracted both viruses from her husband. Only two male patients gave a history of occasional male sexual partners.

The summary of lab parameters is given in Table 2.

**Table 2:** Laboratory parameters at the time of presentation ALT: alanine transaminase; AST: aspartate aminotransferase

	Mean (range)	
	Non-cirrhotic	Cirrhotic
Bilirubin (T)	0.8 (0.2-2.1) mg/dL	2.8 (1.2-24.2) mg/dL
Bilirubin (D)	0.3 (0.1-1.1) mg/dL	1.8 (0.4-16.8) mg/dL
ALT	88 (15-210) IU/mL	91 (16-128) IU/mL
AST	72 (15-208) IU/mL	74 (19-250) IU/mL
Albumin	3.7 (3.0-4.1) mg/dL	2.6 (2.1-4.0) mg/dL
Hemoglobin	15 (12.4- 20.2) gm/dL	12.8 (12-14.2) gm/dL
Platelets (X10^3^)	232 (148-535)/mm^3^	122 (88-132)/mm^3^
Creatinine	0.6 (0.2-1.0) mg/dL	0.7 (0.2-1.2) mg/dL

**Figure 1:**
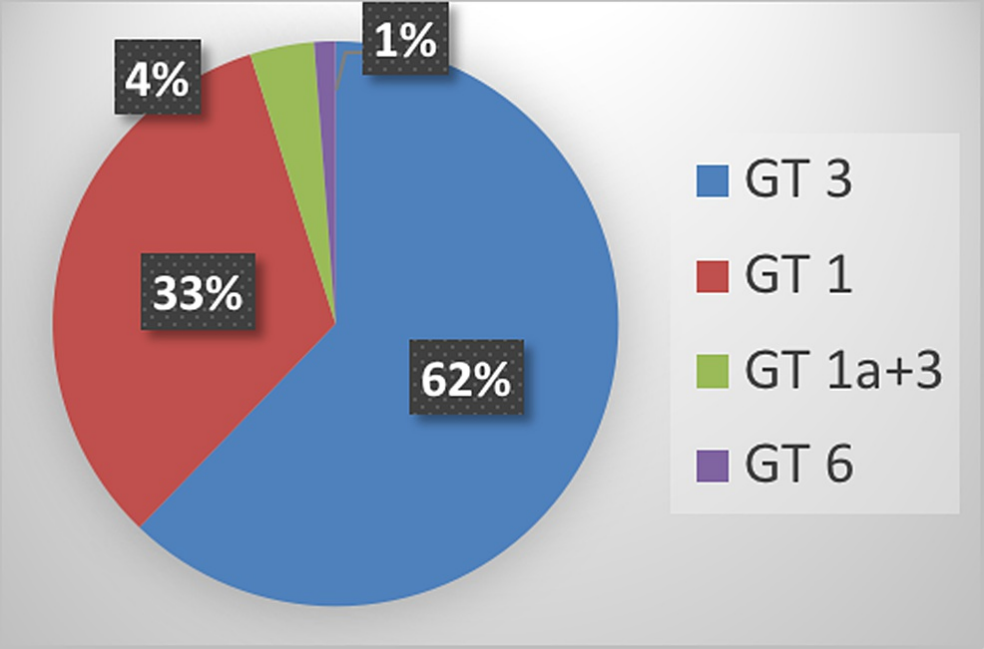
Genotype distribution

Patients were grouped into the cirrhotic and non-cirrhotic groups. Spearman correlation between aspartate aminotransferase to platelet ratio index (APRI) and fibroscan score was seen. Spearman's correlation coefficient was positive with an r-value of 0.78 (Figure 1), and this is statistically significant. There were 29 (35%) cirrhotic patients at presentation. Except for two patients, all were compensated. Both of the decompensated LC had GT 3 disease.

**Figure 2:**
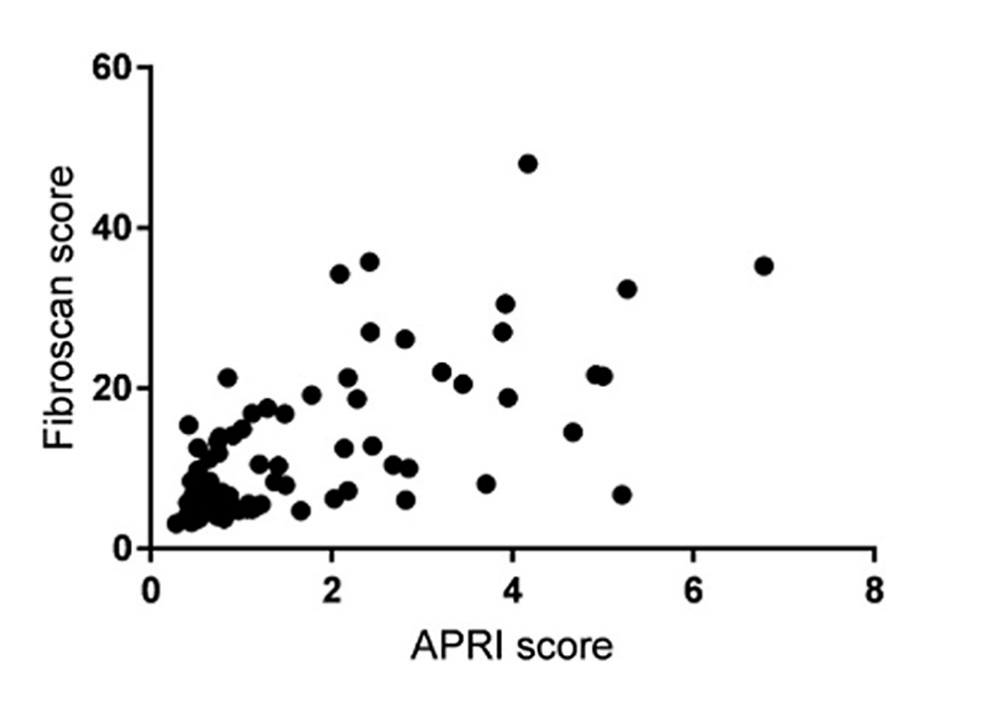
Correlation between fibroscan score and APRI score APRI: aspartate aminotransferase to platelet ratio index

There was a predominance of GT 3. Fifty-one (62%) patients were GT 3, 27 (33%) were GT 1, three (4%) were mixed 1/3, and one (1%) was 6 (Figure 2).

It was predominantly 3a and 1a among GT 3 and 1. The HCV viral load is given in Table 3.

**Table 3:** HCV viral load HCV: hepatitis C virus

HCV VL range (IU/mL)	n	%
16 – 800,000	35	43%
800,000 – 1,000,000	37	45%
>1000000	10	12%

Forty-three percent (43%) patients had a low viral load while 12% of patients had a very high viral load. The ARV regimen before the initiation of treatment is given in Table 1. Twenty-two patients needed to switch over from nevirapine to efavirenz/atazanavir. Most of the patients were having undetectable HIV virus at the time of presentation, as the mean duration of ART was three years five months.

A total of 74 (90%) had SVR12. Non-cirrhotics had 96% SVR compared to 79% in cirrhotics. SVR in GT 3 was 88% while it was 93% in GT 1. These results are comparable with the results of treatment of non-cirrhotic mono-infected individuals (data not shown). There was no correlation between viral load and SVR. High fibroscan value and high APRI were associated with no SVR. The mean value was higher in the non-SVR group, and it was statistically significant (Tables 4-5).

**Table 4:** Correlation between baseline fibroscan score and SVR SVR: sustained virological response

SVR	Mean	Std Dev	SE of Mean	Mean Difference	t	P-Value
No	21.50	10.06	3.56	2.546	3.201	0.002*
Yes	10.99	8.69	1.01			

**Table 5:** Correlation between APRI score and SVR APRI: aspartate aminotransferase to platelet ratio index; SVR: sustained virological response

SVR	Mean	Std Dev	SE of Mean	Mean Difference	t	P-Value
No	2.85	1.63	0.58	0.418	2.56	0.012*
Yes	1.52	1.37	0.16			

The side effects developed during treatments were not life-threatening. There were three deaths during the study. Those deaths were due to complications developed after gall stone pancreatitis. One patient gave a history of binge drinking before admission. Five compensated LC patients developed ascites after one month of treatment. However, they were managed with diuretic therapy and fluid restrictions. Six patients had a fall in hemoglobin after taking ribavirin. It was managed with dose reduction and oral iron therapy. Toward the end of the treatment, all had hemoglobin > 10 mg/dL. Four patients developed jaundice after changes of nevirapine to atazanavir. However, there was no rise in liver enzymes on serial follow-up and the jaundice was predominantly unconjugated hyperbilirubinemia; no intervention was done. The side effects are listed in Table 6.

**Table 6:** Adverse effects during treatment

Adverse effects	n	%
Rise in uric acid	26	32
Gastric upsets	22	27
Fatigue	18	22
Thrombocytopenia	10	12
Myalgia	8	10
Anemia (fall below 10 gm/dL)	6	7
Ascites	5	6

## Discussion

With the availability of highly effective DAAs, global HCV elimination is a burning topic. Will we be able to eradicate HCV from the globe by 2030 is the million-dollar question. HIV/HCV co-infected patients were regarded as difficult to treat patients in the interferon era. However, our study result showed that these patients are no more difficult to treat with the advent of new DAAs.

There was a male preponderance in our study, as only 5% of the total population were females and the rest 95% were males. This is in tune with a previous study [15].^ ^It clearly showed that IV drug abuse is less common among females in the Nepali community. The mode of transmission was the exchange of needles in these patients, making this disease specific to the disease caused by addiction and self-inflicted injury. It was interesting to note that all but two patients had tattoos in different parts of the body, showing a special affinity of the drug abusers’ community towards tattooing. Eighty percent of patients were from the 20-40 years age group, showing that HCV disease is a disease of younger age groups in Nepal. The problem of males having sex with men is less common in Nepal, as only two patients showed this behavior.

Genotype 3 is the most prevalent GT in Nepal. It was 59.8% in one study from Nepal [15]. A similar result was seen in this study, and GT 3 was seen in 62% among co-infected patients, showing no change in its distribution. GT 1 is another GT prevalent, with 33% involvement. A single patient with GT 6 had contracted the disease while he was staying in Hongkong. For the first time, we found mixed GT in Nepal. It was seen in 4% of patients.

The correlation of APRI, fibrosis, and fibroscan have been studied in monoinfected CHC patients [16-17]. However, such a study to compare and correlate APRI and fibroscan has not been explored in co-infected patients in past studies. In this study, an attempt was made to demonstrate the correlation between APRI and fibroscan. There was a significant correlation as evident by Spearman’s correlation, which was 0.78. Thus, APRI can be used as a surrogate marker of fibrosis if a biopsy is not possible and fibroscan is not available. This will help in treating patients in resource-limited settings.

Although there were three deaths during the study, those were not directly related to DAA-related adverse events. All three patients were cirrhotic and were diagnosed to have gall stone pancreatitis. Pancreatitis after the initiation of DAAs is not reported so far. However, there have been case reports regarding the development of pancreatitis after taking ribavirine [18]. In our study, all three patients had gall stones and its role can’t be ignored. Moreover, one patient gave a history of binge drinking. Otherwise compensated before treatment, five patients decompensated after the initiation of treatment but were managed easily without further complications. Other side effects of DAAs and ribavirine were managed easily. There was no drop-out during the study.

It is now well-documented that HIV infection accelerates HCV-associated liver fibrosis, most notably in those with more advanced immunodeficiency. It results in high rates of end-stage liver disease, and survival is shortened after hepatic decompensation events [19-20]. Despite significantly improved life expectancy in HIV infection, liver disease remains a major non-AIDS cause of mortality among HIV/HCV co-infected patients [21]. Thus, effective treatment of HCV in HIV-HCV coinfection is a public health priority, particularly for those with more advanced HCV or HIV disease. Real-life experience showed that the DAAs are equally effective in HCV/HIV co-infected patients. In non-cirrhotic patients, the result is comparable to mono-infected patients. Now it’s time to integrate HIV patients into HCV trials so that more patients can be benefited.

Genotype 3 co-infected are also difficult-to-treat patients. It was also true for HCV/HIV co-infection. SVR in GT 3 was 88% while it was 93% in GT 1. When compared to a similar number of mono-infected individuals in the same institute for the same duration, SVR was less. When we analyzed the data, we found that for fibrosis (as evident by the fibroscan score), the mean score was more in co-infected individuals. More fibrosis at the time of presentation may be the cause of decreased SVR in these patients. GT 3 was also associated with two decompensated cirrhosis patients. Out of the five patients who showed signs of decompensation after the initiation of treatment, four were GT 3. Thus, GT 3 was found to be the more difficult-to-treat group as compared to GT 1.

## Conclusions

Nepal already presented remarkable achievements in infectious diseases, particularly the HIV response. WHO has set a goal to eliminate HCV by the year 2030 AD. With the availability of generic DAAs and acceptable SVR in mono and co-infected patients, we may attain the goal set by WHO. As the target group is defined (IV drug abusers), we can identify, screen, and treat those individuals.
